# Multi-omics approach to reveal follicular metabolic changes and their effects on oocyte competence in PCOS patients

**DOI:** 10.3389/fendo.2024.1426517

**Published:** 2024-10-11

**Authors:** Yuezhou Chen, Minyu Xie, Siyun Wu, Zehua Deng, Yan Tang, Yiqing Guan, Yun Ye, Qiandong He, Lei Li

**Affiliations:** ^1^ Center for Reproductive Medicine, Zhongshan City People’s Hospital, Zhongshan, China; ^2^ Department of Obstetrics and Gynecology, Center for Reproductive Medicine, The Third Affiliated Hospital, Guangzhou Medical University, Guangzhou, China; ^3^ Guangdong Provincial Key Laboratory of Major Obstetric Diseases, The Third Affiliated Hospital, Guangzhou Medical University, Guangzhou, China; ^4^ Guangdong Provincial Clinical Research Center for Obstetrics and Gynecology, The Third Affiliated Hospital, Guangzhou Medical University, Guangzhou, China; ^5^ Guangdong-Hong Kong-Macao Greater Bay Area Higher Education Joint Laboratory of Maternal-Fetal Medicine, The Third Affiliated Hospital, Guangzhou Medical University, Guangzhou, China; ^6^ Key Laboratory for Reproductive Medicine of Guangdong Province, Guangzhou Medical University, Guangzhou, China

**Keywords:** polycystic ovary syndrome, follicle fluid, cumulus cells, transcriptomics, metabolomics, oocyte competence

## Abstract

**Background:**

Polycystic ovary syndrome (PCOS) is a common heterogeneous disorder linked with endocrine and metabolic disturbances. The underlying mechanism of PCOS, especially its effect on oocyte competence, remains unclear. The study aimed to identify abnormal follicular metabolic changes using a multi-omics approach in follicular fluid from PCOS patients and to determine their effects on oocyte competence.

**Methods:**

A total of 36 women with PCOS and 35 women without PCOS who underwent *in vitro* fertilization and embryo transfer were included in the study. Cumulus cells and follicular fluid samples were collected. Follicular fluid samples underwent metabolomic analysis, while cumulus cell clusters from the same patients were assessed using transcriptomic analysis. Clinical information of patients and assisted reproductive technology (ART) results were recorded. Transcriptomics and metabolomics were integrated to identify disrupted pathways, and receiver operation characteristics (ROC) analysis was conducted to identify potential diagnostic biomarkers for PCOS. Pearson correlation analysis was conducted to assess the relationship between metabolites in follicular fluid and oocyte competence (fertilization and early embryo development potential).

**Results:**

Through multi-omics analysis, we identified aberrantly expressed pathways at both transcriptional and metabolic levels, such as the citrate cycle (TCA cycle), oxidative phosphorylation, the cAMP signaling pathway, the mTOR signaling pathway, and steroid hormone biosynthesis. Ten candidate metabolites were identified based on metabolic profiling data from these altered pathways. Phytic acid, succinic acid, 2’-deoxyinosine triphosphate, and 4-trimethylammoniobutanoic acid in the follicular fluid exhibited high specificity and sensitivity in distinguishing PCOS. Among these metabolites, L-arginine showed a negative correlation with the 2PN fertilization rate and cleavage rate, while estrone sulfate showed a negative correlation with the high-quality embryo rate in the *in-vitro* fertilization (IVF) cycle.

**Conclusions:**

We have conducted a preliminary study of a novel metabolic signature in women with PCOS using a multi-omics approach. The alterations in key metabolic pathways may enhance our understanding of the pathogenesis of PCOS.

## 1 Introduction

Polycystic ovary syndrome (PCOS) is a prevalent heterogeneous disease worldwide, associated with endocrine and metabolic disorders, affecting approximately 5-10% of women of reproductive age (1). Hyperandrogenism, ovulatory dysfunction, polycystic ovaries detected via ultrasound, insulin resistance (IR), and obesity are common clinical features of PCOS (2). Patients with PCOS may produce morphologically normal metaphase II oocytes during *in vitro* fertilization and embryo transfer (IVF-ET) procedures, with alterations in oocyte competence being considered potential causative factors for infertility (3). While generally attributed to interactions among genetic, environmental, metabolic, neuroendocrine, and lifestyle factors, the underlying mechanism of PCOS, particularly its impact on oocyte competence, remains unclear (4, 5).

The microenvironment for oocyte development includes follicular fluid (FF), cumulus cells (CCs), granulosa cells (GCs), and oocytes (6). FF contains metabolites from various metabolic processes during oocyte and follicle development, which further support the growth of oocytes and follicles (7). Consequently, metabolic products in FF may have a more direct impact on oocyte quality compared to those in serum and urine (8). Analyzing metabolic changes in PCOS FF may not only aid in understanding pathology but also uncover the influence of metabolites on oocyte competence (9, 10). Some research has focused on identifying metabolic biomarkers and exploring the pathophysiological mechanisms of PCOS (9). Metabolomic studies in FF have identified imbalanced redox potential and increased oxidative stress as potential factors resulting from or contributing to PCOS (8). Dysfunctions in glucose, lipid, and amino acid metabolism might play an important role in the development of PCOS as revealed by metabolomic analysis (9). Certain metabolites, such as trilauric glyceride, decanoylcarnitine, homocysteine and 7β-Hydroxycholesterol, have been investigated as potential biomarkers and may contribute to impaired oocyte competence in PCOS patients (11, 12). Despite providing important information about changes in metabolites, uncertainty remains regarding the specificity of metabolomic biomarkers and pathways when using metabolomics alone (9). Therefore, complementary and surrogate approaches are still necessary to further confirm critical pathways in the pathogenesis of PCOS.

CCs, which surround the oocyte, originate from relatively undifferentiated GCs. The crucial roles of CCs in supporting follicular development have prompted numerous researchers to concentrate on this unique cluster of cells (13). Extensive investigation of genes in CCs and GCs has yielded new evidence for understanding the pathogenesis of PCOS (14, 15). CCs from PCOS patients exhibit abnormal gene expression, including dysregulated growth factors, cell cycle regulation, inflammatory function, metabolic pathways, and oxidative phosphorylation (14). Moreover, aberrant expression of miRNA and lncRNA in granulosa cells might contribute to the development of PCOS (16). Despite the generation of extensive gene expression data from various studies on the CCs transcriptome (17–19), the variability in study endpoints makes it challenging to link different pathways or mechanisms. Nonetheless, gene expression profiling obtained from transcriptomic research has shown significant diagnostic value and potential to elucidate the pathogenesis of PCOS.

A multi-omics approach could offer a method for identifying potential biomarkers and understanding the pathological mechanisms of diseases (16, 20–22). Our objective is to identify metabolic biomarkers using a multi-omics approach that are correlated with the pathological mechanisms of PCOS and compromised oocyte competence in IVF cycles. In this study, we conducted an untargeted metabolomic analysis of follicular fluid samples from patients with PCOS and controls without PCOS using liquid chromatography-mass spectrometry (LC-MS). Our aim was to identify key metabolic alterations unique to PCOS. Subsequently, we conducted transcriptomic analysis on corresponding cumulus cell (CC) samples to identify differentially expressed genes (DEGs). Finally, we integrated our metabolomic and transcriptomic data to elucidate disrupted pathways at both metabolic and transcriptional levels and identify potential diagnostic biomarkers for PCOS.

## 2 Methods

### 2.1 Human subjects

Seventy-one women, aged 22-37 years, undergoing IVF, were recruited for this study. Among them, 36 patients had PCOS, and 35 controls did not. PCOS was diagnosed according to the revised 2003 Rotterdam criteria, which necessitate the presence of two out of three indicators: oligo- or anovulation, signs of clinical and/or biochemical hyperandrogenism, and polycystic ovaries on ultrasonography after excluding other etiologies (such as androgen-secreting tumors, congenital adrenal hyperplasia, and Cushing’s syndrome). The control group subjects had regular menstrual cycles, no signs of hyperandrogenism, and normal ovarian morphology as determined by ultrasonography. The 35 controls were recruited from women with serum FSH levels < 10IU/L and AMH levels > 1.5ng/mL on day 2 or 3 of the menstrual cycle, who participated in IVF-ET treatment due to fallopian tubal obstruction. To ensure ovarian reactivity in the control group, a follicle count of more than 5 was required on the day of human chorionic gonadotropin (HCG) trigger. Informed consent was obtained from all study participants. The study received approval from the local Ethics Committee of the Third Affiliated Hospital of Guangzhou Medical University (No. 2023-150).

### 2.2 Ovarian stimulation and samples collection

All subjects underwent controlled ovarian hyperstimulation following our established protocols. When more than three follicles reached a diameter larger than 18 mm, human chorionic gonadotropin (hCG) was administered intramuscularly. Oocyte retrieval was conducted 36 hours after hCG injection using transvaginal ultrasound-guided needle puncture for follicles with a diameter greater than 14 mm. CCs were mechanically dissected from the oocytes under stereomicroscopy using a glass pipette with an inner diameter of 200 μm. CCs from each patient were pooled. The recovered CCs were washed with 1 ml of phosphate-buffered saline (PBS) and flash-frozen in liquid nitrogen. The CC samples were stored at -80°C until needed. After oocytes collecting, the follicular fluid paired with CCs, free from contaminating blood from the same patient, was combined and centrifuged at 12,000 rpm for 10 minutes. The supernatant was transferred to a 1.5 ml EP tube and stored at -80°C until analysis.

### 2.3 Embryo assessment

Oocytes were inseminated with spermatozoa prepared by gradient centrifugation approximately 4-6 hours after follicle aspiration. Normal diploid fertilization was assessed 16-18 hours after insemination. Only oocytes displaying two visible pronuclei (2PN) were considered normal. The 2PN fertilization rate was calculated as the ratio of the number of oocytes with 2PN to the total number of inseminated oocytes. The cleavage rate was calculated as the ratio of the number of cleaved embryos to the number of oocytes with 2PN. Embryo quality on day 3 was assessed 68 hours after insemination. High-quality embryos on day 3, resulting from normal fertilization of oocytes, consisted of 6-8 cells, exhibited good cell-cell contact, were free from multinucleation, and had less than 10% volume fragmentation. The high-quality embryo rate was calculated by dividing the number of high-quality embryos by the 2PN cleavage number.

### 2.4 UPLC-MS/MS detection

Ultra-performance liquid chromatography/tandem mass spectrometry (UPLC-MS/MS) analysis was conducted by Oebiotech Co., Ltd. (Shanghai, China). Briefly, 100 μl of samples were mixed with 10 μl of L-2-chlorophenylalanine (0.3 mg/ml) dissolved in methanol as the internal standard in a 1.5 ml EP tube, followed by vortexing for 10 seconds. Then, 300 μl of an ice-cold mixture of methanol and acetonitrile (2/1, vol/vol) was added, and the mixture was vortexed for 1 minute, followed by ultrasonication for 10 minutes in an ice-water bath. The extract was centrifuged at 13,000 rpm for 10 minutes at 4°C. The supernatants from each tube were filtered through 0.22 μm microfilters. Quality control (QC) samples were prepared by mixing aliquots of all samples to create a pooled sample. Metabolic profiling was performed using an ACQUITY UPLC BEH C18 (1.7 μm, 100×2.1 mm) system (Waters, Milford, USA) in both ESI positive and ESI negative ion modes. Mobile phases A and B consisted of water and acetonitrile/methanol (2/3, v/v), both containing 0.1% formic acid. The flow rate was 0.4 ml/min and column temperature was 45°C. The injection volume was 1 μl. Mass detection was performed using a QE-HF mass spectrum detector (Thermo Fisher Scientific, Waltham, USA) with mass spectrophotometer scanning. Data acquisition was conducted in both full scan mode (50-1000 m/z) and MSE mode. Metabolites were identified using Progenesis QI data processing software (Waters, Milford, USA). Principal component analysis (PCA) and (orthogonal) partial least squares-discriminant analysis (OPLS-DA) were performed to visualize the metabolic differences between groups. The metabolites with variable importance of projection (VIP) >1 and *P*< 0.05 were considered as differential metabolites.

### 2.5 Transcriptomic sequencing

Total RNA from CCs was extracted using the TRIzol Reagent Kit (Life Technologies, Carlsbad, USA) according to the standard protocol. RNA quality was assessed using a Nanophotometer spectrophotometer (IMPLEN, CA, USA) with 1% agarose gels. Total RNA was then purified using the RNeasy Mini Kit (QIAGEN, GmBH, Germany) and treated with the RNase-Free DNase Set (QIAGEN, GmBH, Germany) to minimize genomic contamination. mRNA was isolated from total RNA using poly-T oligo-attached magnetic beads and ribosomal RNA was removed. The harvested mRNA was fragmented randomly using divalent cations in the NEB fragmentation buffer. The first strand cDNA was synthesized from 3 μg of RNA using random hexamer primers and M-MuLV Reverse Transcriptase (RNase H). PCR was performed using Phusion High-Fidelity DNA polymerase, universal PCR primers, and Index (X) Primer. PCR products were purified using the AMPure XP system. The sequencing library was prepared using the RNA Library Prep Kit, diluted to 10 pM, and sequenced on the HiSeq X Ten (Illumina, USA) platform at Novogene Science and Technology Co., Ltd. (Beijing, China). Clean reads were obtained by filtering out reads containing adapters, poly-N sequences, and low-quality reads from the raw data. FPKM of each gene were calculated based on the gene length and the number of reads mapped to that gene. Differentially expressed genes (DEGs) were analyzed using the DESeq2 R package (version 1.28.1, http://www.bioconductor.org/packages/release/bioc/html/DESeq2.html). Gene Ontology (GO) and Kyoto Encyclopedia of Genes and Genomes (KEGG) pathway analyses were conducted using the R package (version 3.16.1, http://www.bioconductor.org/packages/release/bioc/html/clusterProfiler.html) to assess the statistical enrichment of DEGs.

### 2.6 Statistical analysis

Statistical analysis was conducted using SPSS 25.0 (SPSS, Chicago, IL, USA). Continuous variables with a normal distribution were expressed as mean ± standard error of the mean (SEM) and analyzed using Student’s t-test. The frequency (composition ratio) of categorical data was statistically described and evaluated using the Chi-square test. Pearson correlation coefficient was utilized for correlation analyses. Statistical significance was defined as *P*< 0.05. Receiver operating characteristic (ROC) curves were fitted to identify candidate metabolites for discriminating PCOS based on the area under the curve (AUC).

## 3 Results

### 3.1 Clinical characteristics

A total of 71 participants, with 36 in the PCOS group and 35 in the control group, were included in this study. The clinical information and assisted reproductive technology (ART) results are presented in **Table 1**. There were no significant differences in age, body mass index (BMI), follicle stimulating hormone (FSH), total testosterone (TT), serum total cholesterol (TC), or fasting glucose levels between the PCOS group and the control group. Antral follicle count (AFC), anti-Mullerian hormone (AMH), luteinizing hormone (LH), LH/FSH ratio, and triglyceride (TG) levels were significantly higher in patients with PCOS compared to controls (*P*< 0.05). There were no statistically significant differences in the number of retrieved oocytes, 2PN fertilization rate, or cleavage rate between the two groups. The high-quality embryo rate in PCOS patients was significantly lower than that in the controls (*P*< 0.05).

**Table 1:** Clinical information and ART results of PCOS patients and control subjects.

	Control (n=35)	PCOS (n=36)	*P*-value
**Age (years)**	30.77 ± 0.64	31.77 ± 0.51	0.631
**BMI(kg/m^2^)**	22.68 ± 0.62	23.04 ± 0.47	0.642
**AFC**	22.23 ± 1.41	29.53 ± 1.79	0.002
**AMH (ng/ml)**	4.96 ± 0.40	7.75 ± 0.77	0.002
**FSH (U/L)**	7.14 ± 1.79	7.47 ± 0.50	0.574
**LH (U/L)**	2.90 ± 0.43	5.77 ± 0.81	0.003
**LH/FSH**	0.46 ± 0.08	0.89 ± 0.13	0.008
**TT (nmol/L)**	1.16 ± 0.09	1.40 ± 0.10	0.073
**TC (nmol/L)**	4.63 ± 0.16	4.93 ± 0.14	0.153
**TG (nmol/L)**	0.99 ± 0.08	1.32 ± 0.11	0.017
**Fasting glucose(mmol/L)**	5.02 ± 0.08	5.02 ± 0.07	0.984
**No. of oocyte retrieve**	16.83 ± 1.02	17.39 ± 1.54	0.764
**2PN fertilization rate (%)**	53.26 (310/582)	54.72 (336/614)	0.613
**Cleavage rate (%)**	96.45 (299/310)	97.02 (326/336)	0.682
**High-quality embryo rate (%)**	38.13 (114/299)	23.90 (78/326)	0.000

Data are presented as means ± SEM. BMI, body mass index; AFC, antral follicle count; AMH, anti-Mullerian hormone; FSH, follicle stimulating hormone; LH, luteinizing hormone; TT, total testosterone; TC, serum total cholesterol; TG, triglyceride; 2PN, two visible pronuclei.

### 3.2 Identification of different metabolites and pathways associated with PCOS

**Figure 1:**
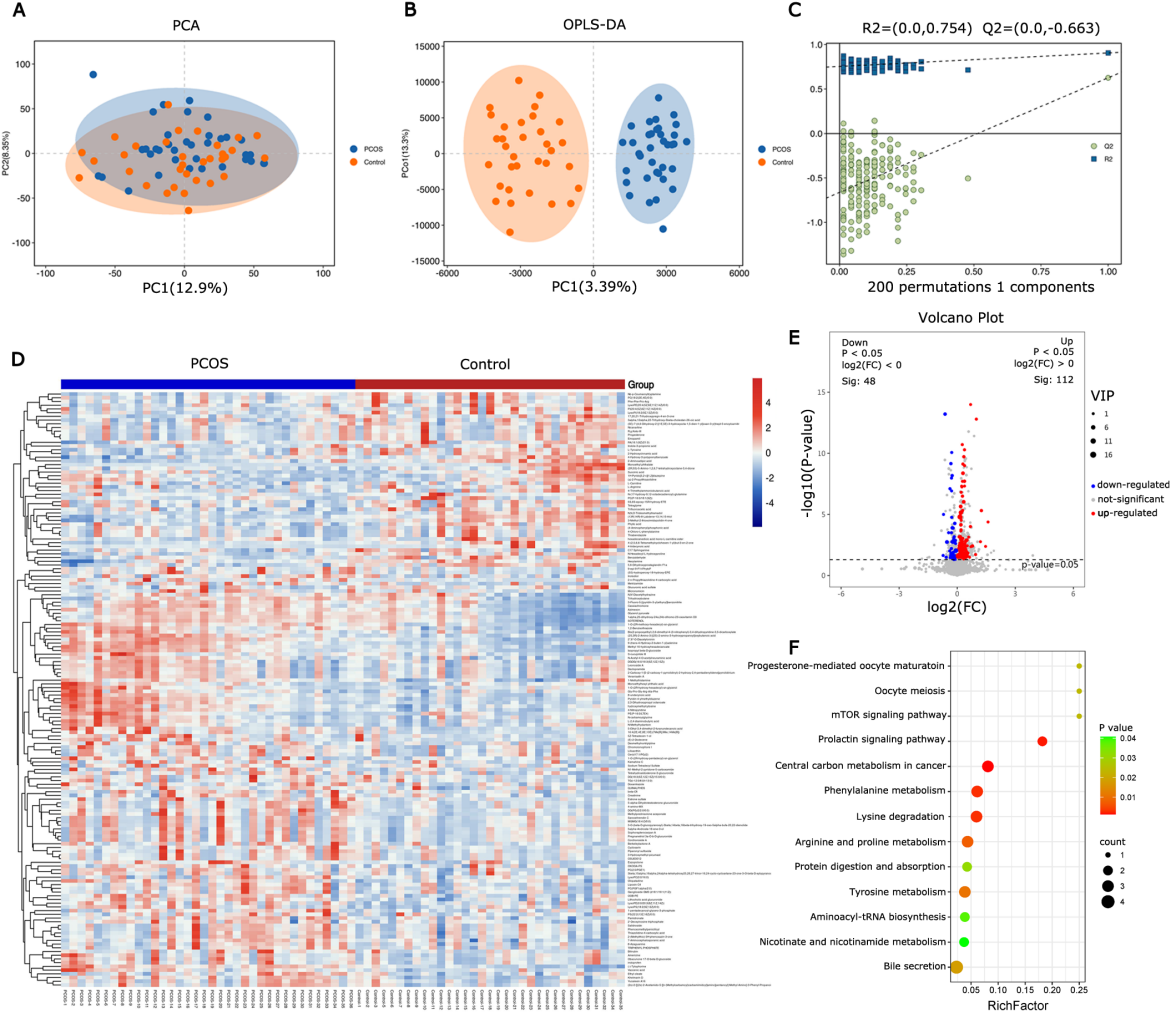
Metabolite and pathway changes in PCOS follicular fluid. **(A)** PCA scores plot and **(B)** orthogonal projections to latent structures-discriminant analysis (OPLS-DA) scores plot based on the metabolic profiling data obtained from follicular fluid of PCOS patients and control women. **(C)** OPLS-DA model was constructed and verified by the permutation test (R^2^ = 0.754, Q^2^ = -0.663). **(D)** Heatmap of differential metabolites from follicular fluid between PCOS and control. **(E)** Volcano map of differential metabolites. **(F)** Bubble chart of enriched KEGG pathway for the differential metabolites in follicular fluid from PCOS and control.

Non-targeted metabolic profiling of FF using UPLC-MS was conducted to provide an overview of metabolic changes in PCOS. The PCA and OPLS-DA models were employed to compare the metabolomic profiles of FF and assess the extent of diversity between PCOS patients and controls (**Figure 1A**). The PCA analysis revealed that this model could not distinguish between the control and PCOS groups. Supervised OPLS-DA was applied to the dataset to maximize group separation and identify discriminating metabolites, while eliminating factors unrelated to group differences. The OPLS-DA model resulted in a clearly discrimination between PCOS patients and healthy controls (R2X(cum) = 0.508, Q2(cum) = 0.626, **Figure 1B**). To prevent overfitting of the model, 7-fold cross-validation and 200 response permutation tests (RPT) were conducted to assess the model’s quality. With Q2 intercepting the y-axis at -0.662, the supervised model was effectively protected against overfitting (**Figure 1C**). The OPLS-DA results revealed significant metabolic differences between PCOS patients and healthy controls.

Hierarchical clustering was employed to analyze the expression levels of all significantly different metabolites (**Figure 1D**). Differential metabolites were screened using VIP≥ 1 and *t*-test *P*< 0.05 as the screening criteria. A total of 160 differential metabolites were identified between PCOS patients and controls, with 112 up-regulated and 48 down-regulated in PCOS follicular fluid (**Figure 1E**). KEGG analysis was conducted on the different metabolites, and metabolic pathways with a P value < 0.05 were selected. The results indicated that the metabolic pathways were predominantly enriched in amino acid metabolism and carbohydrate metabolism (**Figure 1F**).

### 3.3 Screening of DEGs in CCs and its functional analysis

**Figure 2:**
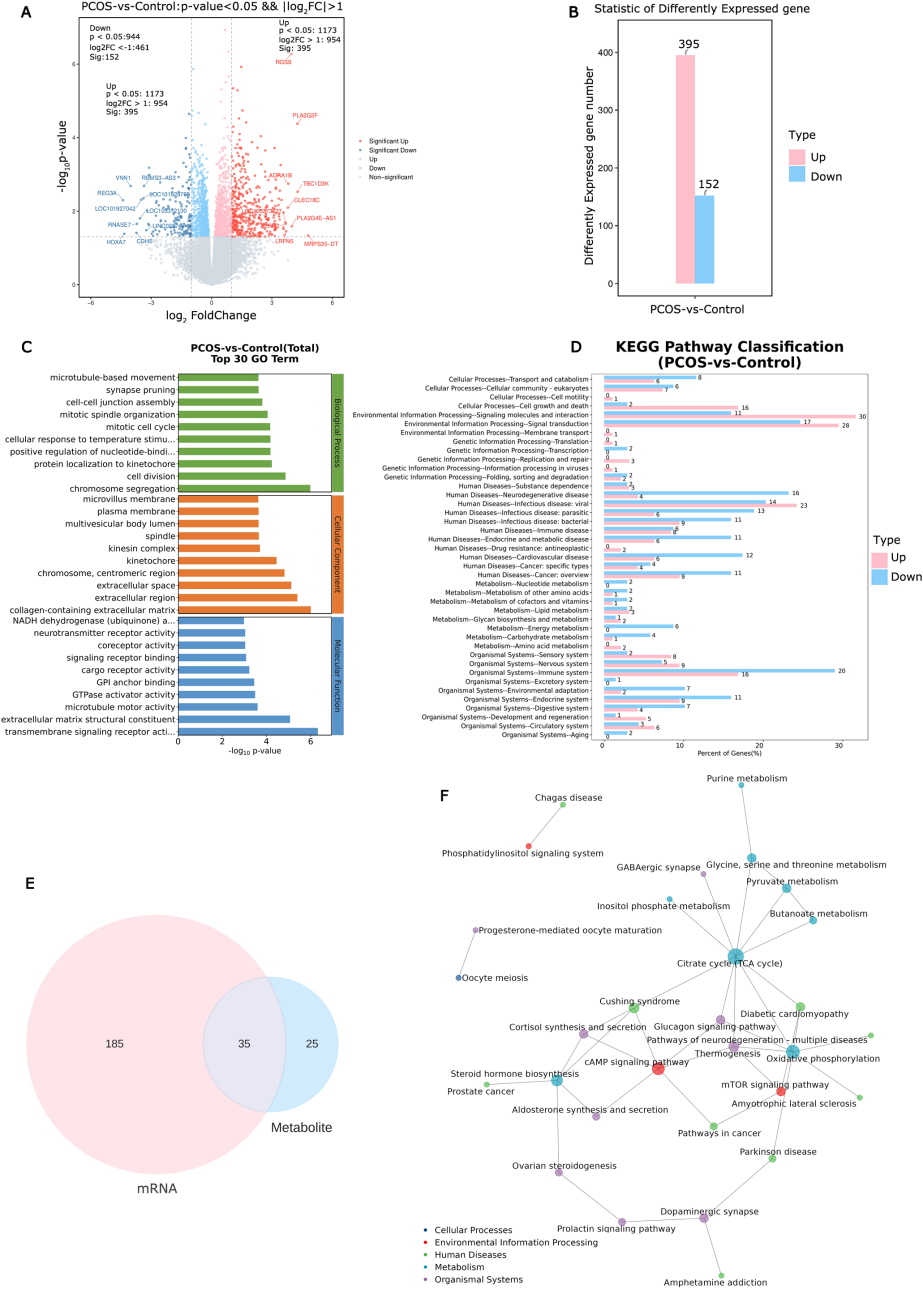
Identification and analysis of differential expression genes in cumulus cells and altered pathways in PCOS. **(A)** The volcano plot of differential expression genes in PCOS cumulus cells. **(B)** Statistic results of up- and down-regulated differential expression genes in PCOS cumulus cells. **(C)** Enriched top 30 GO terms of differential expression genes. **(D)** Enriched KEGG pathways of differential expression genes. **(E)** Metabolic and transcriptional analysis identified 35 pathways that were significantly altered in PCOS. **(F)** KGML network building based on the differential expression genes and differential metabolites to obtain the critical metabolism pathways.

Transcriptional profiling of the corresponding cumulus cells (CCs) samples was conducted to trace upstream variations in the metabolome. In CCs of PCOS patients, 547 DEGs ( |log2FC|>1, P<0.05 , 395 up-regulated, 152 down-regulated) were identified compared to the control group (**Figures 2A, B**). Functional analysis of DEGs was conducted using GO and Kyoto Encyclopedia of Genes and Genomes (KEGG) enrichment analyses. The DEGs were co-enriched in 3371 GO terms, with biological process (BP) accounting for 2272/3371, molecular function (MF) accounting for 444/3371, and cell component (CC) 655/3371 (**Figure 2C**). A total of 362 GO terms were significantly enriched (*P*< 0.05). KEGG analysis of DEGs revealed significant enrichment in lipid metabolism, carbohydrate metabolism, amino acid metabolism, and signal transduction pathways (**Figure 2D**).

### 3.4 Association analysis of metabolome and transcriptome reveals the altered pathways in CCs

**Figure 3:**
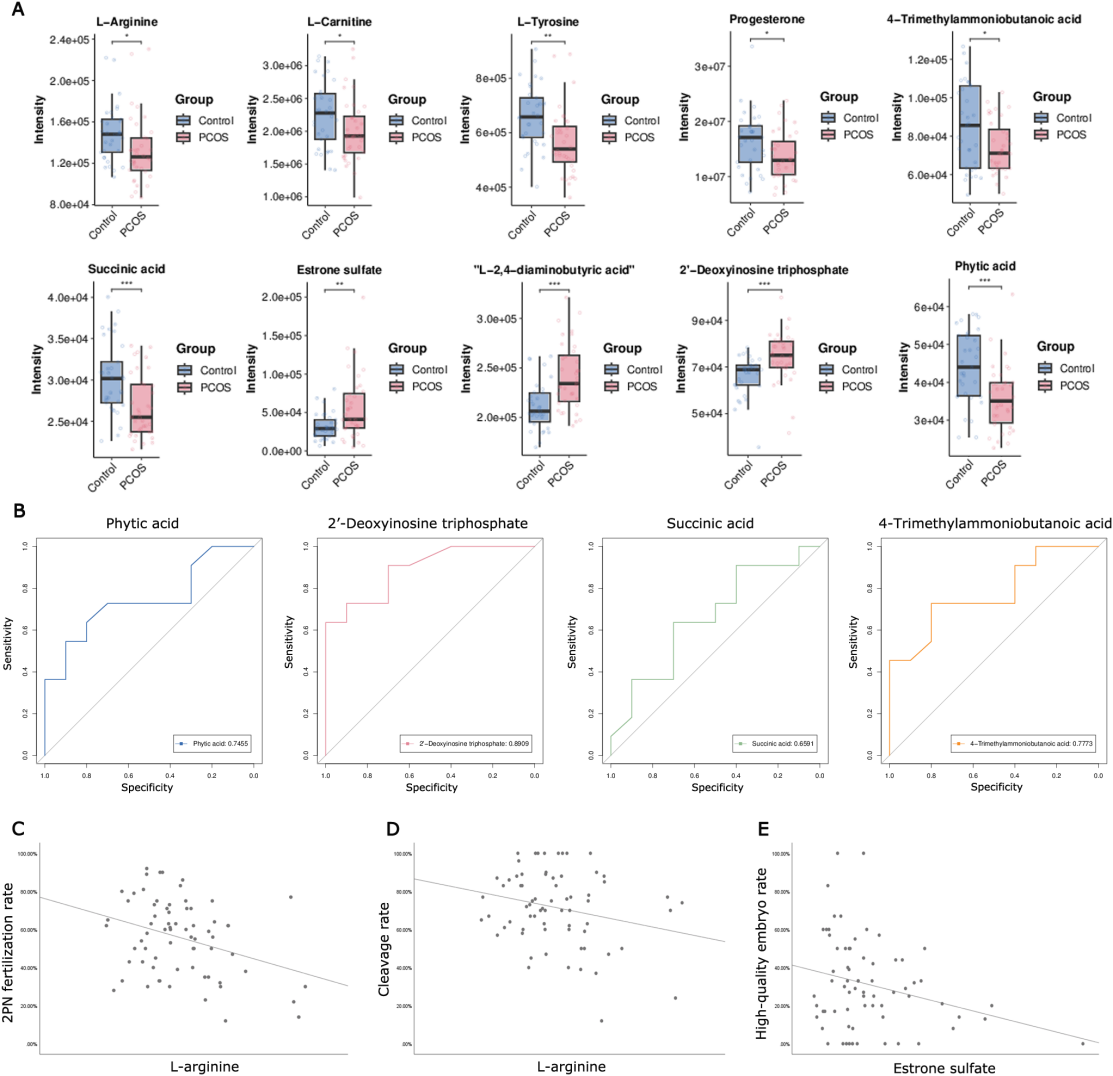
Metabolites analysis based on altered pathways at both the metabolomic and mRNA expression levels, and Pearson correlations analysis between candidate metabolites in FF and the parameters of oocyte competence. **(A)** The changes in relative levels of identified metabolites involved in the significant pathways (^*^ *P*< 0.05; ^**^ *P*< 0.01; ^***^ *P*< 0.001). **(B)** Receiver operating curves (ROC) of potential biomarkers for PCOS. **(C)** Scatter plot of 2PN fertilization rate and intrafollicular L-arginine level (*P*< 0.05). **(D)** Scatter plot of cleavage rate and intrafollicular L-arginine level (*P*< 0.05). **(E)** Scatter plot of high-quality embryo rate and estrone sulfate level (*P*< 0.05).

Metabolic pathway analysis and gene set enrichment analysis revealed 35 significantly altered pathways at both the metabolomic and mRNA expression levels (**Figure 2E**). These pathways include the citrate cycle (TCA cycle), oxidative phosphorylation, cAMP signaling pathway, mammalian target of rapamycin (mTOR) signaling pathway, and steroid hormone biosynthesis (**Figure 2F**). Of the 35 altered pathways, 10 metabolites exhibited significant differences based on metabolic profiling data (*P*< 0.05, **Figure 3A**).

### 3.5 The candidate value of metabolites in FF for PCOS

ROC curve analysis was employed to determine the predictive value of 10 metabolites for PCOS, obtaining the AUC, 95% CI, specificity, and sensitivity. Typically, an area under the ROC (AUC) greater than 0.65 is considered indicative of better predictive performance; therefore, only results with an AUC greater than 0.65 are listed. As depicted in **Figure 3B**, the AUC values for phytic acid, succinic acid, 2’-deoxyinosine triphosphate, and 4-trimethylammoniobutanoic acid were 0.746 (0.525-0.966), 0.659 (0.414-0.904), 0.891 (0.756-1.000), and 0.777 (0.572-0.982), respectively (**Figure 3B**). Considering biological and statistical factors, phytic acid, succinic acid, 2’-deoxyinosine triphosphate, and 4-trimethylammoniobutanoic acid in FF may serve as potential biomarkers of PCOS.

### 3.6 Effects of metabolic perturbation on ART results

To investigate the metabolic effects on ART outcomes, Pearson correlations were calculated between 10 selected metabolites in FF and oocyte competence parameters. L-arginine showed a negative correlation with the 2PN fertilization rate and cleavage rate among these candidate FF metabolites (*P*< 0.05, **Figures 3C, D**). Estrone sulfate exhibited a negative correlation with the high-quality embryo rate in the overall sample cohort (*P*< 0.05, **Figure 3E**).

## 4 Discussion

In this study, we integrated metabolomics and transcriptomics data, identifying 35 significantly altered pathways at both the metabolic and transcriptional levels, including the citrate cycle (TCA cycle), oxidative phosphorylation, cAMP signaling pathway, mTOR signaling pathway, and steroid hormone biosynthesis. Among these altered pathways, we identified 10 metabolites using metabolic profiling data. According to ROC analysis, phytic acid, succinic acid, 2’-deoxyinosine triphosphate, and 4-trimethylammoniobutanoic acid may serve as potential biomarkers of PCOS. Furthermore, we demonstrated that L-arginine and estrone sulfate were closely associated with the 2PN fertilization rate, cleavage rate, and high-quality embryo rate in the IVF cycle.

Metabolomic analysis of FF is a crucial method for elucidating the mechanisms underlying metabolic disorders associated with PCOS (9). Potential biomarkers and various pathways implicated in PCOS include those associated with glucose metabolism, amino acid metabolism, and lipid metabolism (7, 23). However, a single metabolomic change in follicular fluid cannot fully reflect the metabolic activity of specific cells (24). This is primarily because FF contains products from the microenvironment of follicle growth and oocyte development, including cumulus cells, granulosa cells, and oocytes (25). Thus, metabolomic analysis of follicular fluid can only offer limited insights into understanding the underlying pathological mechanism of PCOS. Considering that functional genes play a crucial role in cell metabolism, quantitative analysis of mRNA using high-throughput microarray analysis may also yield significant insights into cell metabolism. Therefore, a combined approach may offer a more comprehensive solution than either approach alone for understanding the pathological mechanisms of diseases (21). In this study, we integrated data from metabolomics and transcriptomics and identified 35 significantly altered pathways, including the citrate cycle (TCA cycle), oxidative phosphorylation, cAMP signaling pathway, mTOR signaling pathway, and steroid hormone biosynthesis. The results provided a comprehensive and updated understanding of the pathogenesis of PCOS.

The TCA cycle plays a central role in cellular metabolism and the regulation of energy homeostasis (26). Inactivation of the TCA cycle results in reduced intracellular levels of ATP and cAMP, thereby affecting the activation of the Ras1-regulated cAMP signaling pathway (27). Studies have identified the TCA cycle and glucose metabolism as the major pathways disrupted in PCOS, which have been proposed as possible links with adipokines (28, 29). Impaired oxidative phosphorylation reduces adenosine triphosphate (ATP) production, potentially contributing to the metabolic and hormonal dysregulation seen in PCOS (30). CCs from PCOS patients were found to have deficiencies in TCA cycle function (8). Succinate is an intermediate in the TCA cycle and plays a crucial role in mitochondrial ATP production. It may impact the developmental potential of oocytes from PCOS patients due to its inflammatory signaling role (31). In our study, the level of succinic acid was significantly reduced in PCOS patients. The decreased level of succinic acid may significantly contribute to the insufficient TCA cycle and abnormal cAMP signaling pathway in PCOS patients.

mTOR is a serine-threonine protein kinase situated downstream of the phosphatidylinositol 3-kinase (PI3K)-Akt pathway (32). This pathway regulates diverse processes, such as cell growth, proliferation, metabolism, and angiogenesis (33). The mTOR signaling cascade has been recently investigated in ovarian follicles, where it controls the proliferation and differentiation of granulosa cells (34). Several studies have proposed that the mTOR signaling system is implicated in the pathophysiological mechanisms of PCOS (35, 36). Overexpression of the mTOR pathway can disrupt cumulus cell interaction, induce insulin resistance, and directly impact follicle growth (37). Abnormalities in mTOR signaling during follicular development result in various pathologies, including premature ovarian insufficiency (POI) and infertility (33). Arginine serves as a direct activator of mTOR and promotes follicular development by stimulating the proliferation of granulosa cells (38). In our study, the arginine level in FF was significantly lower in PCOS women compared to the controls. This result was consistent with previous findings obtained from serum samples of PCOS patients (39). Furthermore, we found that the arginine level in FF correlated with the 2PN fertilization rate and cleavage rate. These findings suggest that the reduced arginine levels in FF might influence follicle development via the mTOR signaling pathway.

PCOS is the most prevalent endocrine disorder affecting female reproductive health, characterized by chronic low-level inflammation and hormone imbalances (40). Inflammation will impair meiotic and cytoplasmic maturation of the oocyte which damages its developmental competence for fertilization and embryo development (41). PCOS patients typically exhibit low levels of progesterone (42). Consequently, the reduced progesterone levels in PCOS lead to immune system overstimulation, thereby increasing estrogen production (40). Estrone sulfate is the predominant estrogen precursor present in the circulation of both women and men (43). Our findings revealed elevated levels of estrone sulfate and significantly reduced progesterone levels in the FF of PCOS patients. These findings align with prior research conducted on plasma samples from PCOS patients (44). Furthermore, estrone sulfate levels in FF exhibited a negative correlation with the rate of high-quality embryos. Discrepancies in estrone sulfate and progesterone levels between the control and PCOS groups suggest their potential involvement in PCOS development and oocyte quality.

PCOS is a complex disorder characterized by a lack of standardized diagnostic criteria, posing challenges for diagnosis (45). Metabolomics, the newest addition to the “omics” family, has been employed to discover potential biomarkers for PCOS (46, 47). Recent studies have identified novel potential FF biomarkers in PCOS patients using a metabolomic approach (11, 48). The ROC curve is commonly used to assess the diagnostic power of biomarkers. In this study, ROC analysis was conducted on differential candidate metabolites to explore their potential as biomarkers for PCOS diagnosis. The results revealed that the AUC values for phytic acid, succinic acid, 2’-deoxyinosine triphosphate, and 4-trimethylammoniobutanoic acid were 0.746, 0.659, 0.891, and 0.777, respectively. Studies have shown that phytic acid possesses antioxidant properties and can scavenge free radicals, which may be detrimental to follicular development (30, 49). 4-trimethylammoniobutanoic acid serves as a precursor to L-carnitine, which plays a crucial role in fatty acid metabolism and their transport across the mitochondrial membrane to the mitochondrial matrix, where β-oxidation of fatty acids (FAs) takes place. Studies have reported that decreased L-carnitine levels could lead to abnormalities in these processes in PCOS, and L-carnitine supplementation might positively impact fertility (9). These findings suggest that phytic acid, along with two other potential biomarkers, may provide valuable diagnostic insights into PCOS.

Although the integration of transcriptomics and metabolomics approaches has provided better insights into metabolic differences in the FF of PCOS and identified correlations between metabolites and ART outcomes, the study has some limitations that need to be addressed. Firstly, the relatively small sample size was a major limitation of the present study, potentially reducing statistical power to detect small changes in the multi-omics profile and occasionally leading to false positive results. Secondly, it is challenging to recruit a control group of patients with proven fertility who have experienced at least one uncomplicated term pregnancy and are undergoing *in vitro* fertilization (IVF) due to male factor infertility. Women with fallopian tube obstruction cannot be regarded as an ideal control group, as they remain part of the infertile population. Furthermore, a significant challenge in clinical sample research involving human matrices is the presence of inter-individual variability, especially pronounced in women with fluctuating hormone levels throughout the menstrual cycle. Despite variations in the ovarian stimulation protocols among study participants, our analysis revealed no statistically significant differences between the two sample groups.

In summary, our study connects alterations in the follicular microenvironment and oocyte development to metabolites in FF and the transcription of functional genes in CCs. Through comprehensive profiling, we identified several candidate metabolites that were altered in the FF of women with PCOS undergoing IVF treatment. These candidate metabolites could serve as potential biomarkers for distinguishing between individuals with PCOS and controls. The changes in these metabolites were closely correlated with the 2PN fertilization rate, cleavage rate, and the rate of high-quality embryos in the IVF cycle. Our findings support the adoption of an integrated multi-omics approach, which could offer deeper insights into understanding the etiology of PCOS and ultimately improve its diagnosis and management.

## Data Availability

RNA-seq data are deposited in the NCBI Gene Expression Omnibus (GEO, accession number GSE277906). Metabolomics data have been deposited in the OMIX, China National Center for Bioinformation / Beijing Institute of Genomics, Chinese Academy of Sciences (https://ngdc.cncb.ac.cn/omix: accession number OMIX007557).
